# Is Two Better than One? Limb Activation Treatment Combined with Contralesional Arm Vibration to Ameliorate Signs of Left Neglect

**DOI:** 10.3389/fnhum.2013.00460

**Published:** 2013-08-07

**Authors:** Marco Pitteri, Giorgio Arcara, Laura Passarini, Francesca Meneghello, Konstantinos Priftis

**Affiliations:** ^1^Laboratory of Neuropsychology, IRCCS San Camillo Hospital, Lido-Venice, Italy; ^2^Department of General Psychology, University of Padova, Padova, Italy

**Keywords:** neglect, rehabilitation, intra-individual variability, repeated measures, limb activation, arm vibration

## Abstract

In the present study, we evaluated the effects of the Limb Activation Treatment (LAT) alone and in combination with the Contralateral Arm Vibration (CAV) on left neglect (LN) rehabilitation. We conceived them as techniques that both prompt the activation of the lesioned right hemisphere because of the activation (with the LAT as an *active* technique) and the stimulation (with the CAV as a *passive* technique) of the left hemibody. To test the effect of the simultaneous use of these two techniques (i.e., LAT and CAV) on visuo-spatial aspects of LN, we described the case of an LN patient (GR), who showed high intra-individual variability (IIV) in performance. Given the high IIV of GR, we used an ABAB repeated-measures design to better define the effectiveness of the combined application of LAT and CAV, as a function of time. The results showed an improvement of GR’s performance on the Bells test following the combined application of LAT and CAV, with respect to the application of LAT alone. We did not find, however, significant effects of treatment on two other LN tests (i.e., Line bisection and Picture scanning). We propose that the combined application of LAT and CAV can be beneficial for some aspects of LN.

## Introduction

One of the major neuropsychological syndromes following right-hemisphere lesion is left neglect (LN). LN patients fail to respond, report, or orient to stimuli in the contralesional left side of space (Heilman et al., 2003). LN comprises a heterogeneous, multifaceted, and highly variable set of behavioral symptoms and signs (Barrett et al., 2006; Adair and Barrett, 2008), which cannot be attributed to primary sensory or motor defects, given that double dissociations have been reported between LN and primary motor and sensory defects (Vallar, 1998). Although some spontaneous recovery occurs in the majority of LN patients after stroke, LN remains severe in many patients and may persist in the chronic phase (Stone et al., 1992; Jehkonen et al., 2000, 2007; Farnè et al., 2004; Rengachary et al., 2011; Nijboer et al., in press). Commonly associated with left hemiplegia, LN renders motor-associated deficits more severe and it is one of the major factors associated with poor functional outcome (Denes et al., 1982; Jehkonen et al., 2001; Buxbaum et al., 2004; Farnè et al., 2004). LN may limit the effectiveness of the rehabilitation interventions, often to a greater extent than more obvious motor, sensory, and speech deficits (Buxbaum et al., 2004). As a consequence, LN contributes to longer time of hospitalization (Katz et al., 1999; Cherney et al., 2001).

In the past decades, the growing of knowledge on the LN syndrome has suggested the implementation of several well-defined, theory-driven LN rehabilitation approaches (for review, see Luauté et al., 2006; Kerkhoff and Schenk, 2012; Riestra and Barrett, 2013). Evidence-based clinical trials that have evaluated the effectiveness of LN rehabilitation treatments are, until now, not sufficient to provide a general consensus for the efficacy of a given LN treatment approach (Riestra and Barrett, 2013). The main reasons for this failure are probably related to the problem of a definition of appropriate measurement criteria for treatment success (Riestra and Barrett, 2013), the limited assessment of LN subtypes (Barrett et al., 2006), and the lack of consideration of intra-individual variability (IIV) of the patients’ performance and their individual complexity (Stuss, 2011).

To take into account the IIV and the individual complexity, several authors have provided evidence of the importance of conducting LN rehabilitation treatments, by using a repeated-measures approach (e.g., Robertson et al., 1998; Samuel et al., 2000; Bailey et al., 2002; Maddicks et al., 2003; Humphreys et al., 2006). In some of these studies, the Limb Activation Treatment (LAT; Robertson and North, 1992) has been used to reduce the visuo-spatial deficits of LN patients both in the acute and in the chronic phase. In a series of studies, Robertson and North (1992, 1993, 1994), and Robertson et al. (1992, 1998) showed that LN signs, on cancelation and reading tasks, decreased significantly when LN patients performed the task while moving their left hand in the left side of space. On the contrary, they showed that the total number of omissions on cancelation tasks did not decrease when one LN patient moved his left hand in the right side of space or his right hand in the left side of space (Robertson and North, 1992). In contrast, reading errors were not reduced by concurrent movements of both hands (Robertson and North, 1994). As a general result, a significant reduction of LN signs occurred only when two conditions were simultaneously accomplished: the active unilateral movement of the left limb (condition 1), took place in the left peripersonal space (condition 2). The same result was observed even when one LN patient could not see his own moving hand (Robertson and North, 1992), suggesting a specific effect of left limb activation, instead of a visual cueing effect, in reducing LN signs. In fact, visual cues have been often reported to reduce LN signs (Riddoch and Humphreys, 1983; Halligan et al., 1991), but they seem not to be as effective as active movements of the left upper limb. Robertson and North (1992), indeed, did not observe any improvement on letter cancelation when the LN patient they tested was instructed to gaze, at regular intervals, toward an irrelevant stimulus placed in the left side of space. Nevertheless, Cubelli et al. (1999) did not find positive effects of LAT in a group study (i.e., only 1 patient out of 10 ameliorated).

Several single-case studies, in which repeated measurements were used, have been reported providing some evidence on the effectiveness of the activation of the contralesional arm in reducing LN signs (e.g., see Bailey et al., 2002; Maddicks et al., 2003). Among the previous studies, Samuel et al. (2000) first reported the possible additive effect of LAT combined with the Visual Scanning Training (VST; Antonucci et al., 1995) in two LN patients, showing that LAT combined with VST may have additive effects to reduce the signs of LN in stroke patients. Nonetheless, these results are far from being clear to speculate on the effectiveness of combining the LAT with the VST. In addition to the single-case and group studies previously discussed, in which *active*, motor-intentional limb activation was used, it is also worth to mention that even *passive* left contralesional upper limb movements can improve LN signs (Frassinetti et al., 2001; Harding and Riddoch, 2009).

The positive effects of LAT reported in some LN patients can be interpreted in terms of the pre-motor theory of spatial attention (Rizzolatti and Camarda, 1987; Rizzolatti and Berti, 1990), for which the attentional and motor circuits are intimately linked in the brain. Thus, by activating the motor circuits of the damaged hemisphere (through the left arm/hand movement), associated attentional circuits in the damaged hemisphere would be recruited, improving the spatial attention orienting to the left side of space. On the bases of the pre-motor theoretical construct, it is possible that the somato-sensory activation in the left side of space through the use of LAT, activates and/or enhances the neural networks that subserve space representation. In fact, if spatial attention is a consequence of the activation of pre-motor neurons, the activation of pre-motor neural circuits of the lesioned hemisphere may improve the conscious perception of stimuli in the contralesional side of space. Therefore, even minimal movements of the contralesional limb, in the contralesional space, might induce sufficient activation of the lesioned hemisphere to reduce the inhibitory competition from the unimpaired hemisphere (Robertson et al., 1998).

Another LN rehabilitation technique is contralesional neck muscles vibration (Karnath et al., 1993; Karnath, 1995; Ferber et al., 1998; Schindler et al., 2002; Johannsen et al., 2003). The discharge induced by vibration of the left neck muscles leads to the “false” interpretation that the left neck muscles have lengthened (Karnath et al., 1993). This observation has been interpreted in terms of neural activation from the neck muscle proprioceptors, particularly from the muscle spindles, of cerebral areas subserving the processing of body-centered coordinates raising from the integration of visuo-spatial and body representational maps. A different interpretation, however, has been proposed by Vallar et al. (1995), who investigated the possibility that left neck muscles stimulation yields unspecific, general activation of the right hemisphere, rather than a selective modulation of the cerebral areas subserving the processing of body-centered coordinates. Vallar et al. studied the effect of unspecific stimulation of the right damaged hemisphere through the use of Transcranial Electrical Nerve Stimulation (TENS) applied on the left, contralesional LN patients’ hemibody. Both the skin and the muscle mechanoreceptors may be stimulated by TENS (Vallar et al., 1995); then the pattern of sensory activation produced by the TENS could not be confined to proprioceptive input only. The stimulation could enhance the proprioceptive input toward the right lesioned hemisphere, given that the stimulation, delivered to the left hemibody, conveys the somato-sensory inputs to the right hemisphere. In contrast with the studies by Karnath et al. (1993) and Karnath (1995), in which no amelioration of LN signs was observed after the contralesional arm vibration (CAV) (used as a control condition), Vallar et al. (1995) showed that the stimulation of the left neck muscles and the stimulation of the dorsal surface of the left hand induced the same improvement of LN patients on a cancelation task, suggesting a role of the unspecific stimulation of the right damaged hemisphere in reducing LN signs.

Combining different rehabilitation methods may increase the effectiveness of cognitive treatments (e.g., Kerkhoff and Schenk, 2012). At least in some cases, there is evidence of the therapeutic effect of the combination of rehabilitation techniques, suggesting that combined treatments may be more effective than single rehabilitation treatments (e.g., Butter and Kirsch, 1992, Experiment 2; Schindler et al., 2002; Schröder et al., 2008; Saevarsson et al., 2010; for review, see Saevarsson et al., 2011). Nonetheless, some studies have reported no better effects of combined treatments with respect to single treatments for LN (e.g., Lafosse et al., 2003; Pizzamiglio et al., 2004; Keller et al., 2009; Polanowska et al., 2009). These findings suggest the need of better studying the combination of multiple treatments on LN rehabilitation, by means of the application of theory-driven cognitive interventions, instead of summing up casually two or more rehabilitation techniques. Probably, one successful way to obtain *additive* positive effects of two or more rehabilitation methods provided simultaneously, is the combination of methods that share a common theoretical framework and, consequently, a common network of neural activation. In fact, the use of cognitive interventions that induce conflicting activation of neural circuits has showed potentially harmful effects (e.g., Keller et al., 2009).

In the present study we tested, for the first time, the combined effect of two techniques: the LAT and the CAV, which have never been used together before for rehabilitation purposes (but see, Karnath, 1995, for a use of contralesional hand vibration as a control experimental task). We decided to evaluate the additive effects of LAT and CAV by using them as techniques that both prompt the enhancement of the right lesioned hemisphere, because of the activation (with the LAT) and the stimulation (with the CAV) of the left upper limb. Indeed, we used the LAT as an *active* limb activation technique (mainly top-down, although a bottom-up component is also present, because of tactile and proprioceptive feedback), whereas we used the CAV as *passive* (i.e., bottom-up) tactile activation technique. To test the possible additive effects of these two techniques (i.e., LAT and CAV) on visuo-spatial aspects of LN, we describe the case of an LN patient (GR) who showed high IIV in his performance. Given the high IIV of GR, we decided to use an ABAB repeated-measures design to better define the effectiveness of the combined application of LAT and CAV, as a function of time. In order to induce the strongest activation of the right lesioned hemisphere, we applied the vibration on the left forearm of the patient, to assure maximal stimulation of the left-forearm mechanoreceptors for maximizing the tactile sensory input toward the right lesioned hemisphere. We expected that the combined application of these two different, but complementary treatments (i.e., LAT and CAV) would be better than the application of only one (i.e., LAT).

## Materials and Methods

### Case description

**Figure 1:**
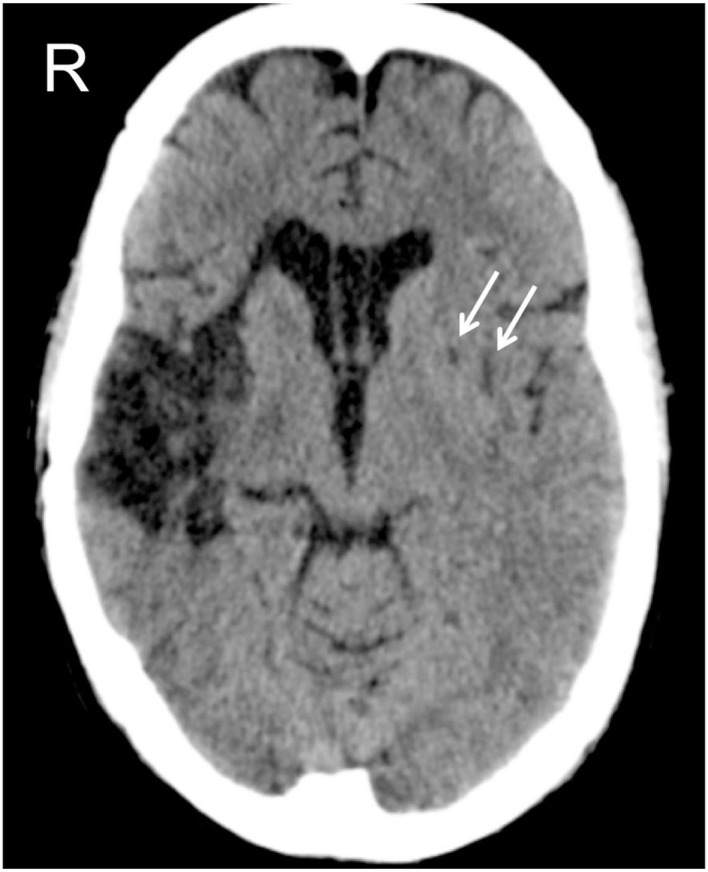
**CAT scan of patient GR, at the level of the basal nuclei**. The right-hemisphere lesion involves the insula, the anterior part of the temporal lobe, and the lenticular nucleus. The left-hemisphere lesion is limited to the lenticular nucleus, indicated by the two white arrows.

GR was a 44-year-old, right-handed man, with 13 years of formal education. GR suffered from hemorrhagic stroke in the right cerebral hemisphere (see Figure 1). As a consequence, GR sustained a neurosurgical intervention, to evacuate the intraparenchymal hematoma. During hospitalization, GR was complied with physical therapy for left hemiparesis and neuropharmacological treatment. GR underwent a formal neuropsychological evaluation 2 months after his right-hemisphere stroke. He was alert and oriented in time, space, and to personal information (Mini Mental State Examination (MMSE) score = 25.2/30, cut-off<24; Magni et al., 1996). GR was unaware of his cognitive and motor defects. GR was collaborative, but he was moderately abulic. Non-spatial cognitive functions, such as memory and language, were intact [*Rey 15*-Item Memory Test (RMT), immediate recall = 28.8/75, cut-off = 28.53; delayed recall = 5.1/15, cut-off = 4.69; Carlesimo et al., 1996 – verbal reasoning equivalent score = 3/4, cut-off = 0; Spinnler and Tognoni, 1987]. Clinical signs of LN, consisting in spontaneous head and gaze deviation toward the ipsilesional (right) side of space, were present. His score on the conventional and behavioral parts of the BIT (Wilson et al., 1987) was below the cut-off (BIT conventional = 27/149, cut-off<130; BIT behavioral = 4/81, cut-off<68), revealing that GR was affected by severe LN, which was exacerbated by the presence of his left homonymous hemianopia.

Because of his severe LN, GR was admitted to an intensive cognitive rehabilitation program (not the one described in the present study) in order to reduce his LN signs. After three months of intensive rehabilitation, and before entering in our study, GR suddenly showed signs of speech apraxia. A CAT scan, performed immediately after the onset of his speech apraxia, revealed a new hemorrhagic stroke in his left cerebral hemisphere (see Figure 1). After 1 month, GR underwent a new formal neuropsychological assessment, which confirmed his preserved non-spatial cognitive abilities (MMSE = 30/30, cut-off<24; Magni et al., 1996 – RMT immediate recall = 51.1/75, cut-off = 28.53; delayed recall = 9.5/15, cut-off = 4.69; Carlesimo et al., 1996 – verbal reasoning equivalent score = 2/4, cut-off = 0; Spinnler and Tognoni, 1987) and the persistence of LN signs (BIT conventional = 100/146, cut-off<130; BIT behavioral = 32/81, cut-off<68; Wilson et al., 1987).

The clinical neuropsychologist who treated GR reported that during the first neuropsychological rehabilitation program (i.e., after his right-hemisphere stroke), GR presented with high IIV of performance on several visuo-spatial tasks (e.g., figure description, drawing completion, etc.). High IIV of performance was also present after his left-hemisphere stroke. The impact of high IIV of GR during cognitive rehabilitation increased the difficulty of performing a comprehensive assessment of the real change achieved through the first rehabilitation program. In fact, a major principle underlying success in cognitive rehabilitation is the capacity of the brain to recover from damage (e.g., Nudo and McNeal, 2013; Sharma et al., 2013), and to re-organize itself in different neural pathways to maximize recovery. Nonetheless, this capacity may not be maximized for the benefit of each patient, because brain plasticity is influenced by many different variables. The success of an intervention, indeed, may not be evident because IIV might not have been appropriately considered. Thus, GR gave his consent to participate in the present rehabilitation study, which started 63 days after the onset of his left-hemisphere stroke. Our goal was to monitor the evolution of his behavioral changes, in order to disentangle his strong IIV in performance from the effects of treatment. GR gave his informed consent to participate in the study, according to the Declaration of Helsinki II.

### Neuropsychological tests

GR was assessed daily, after each cognitive rehabilitation session, through a brief battery of neuropsychological tests for neglect-related disorders in the peripersonal space. The battery included a Line bisection test (i.e., the Line bisection subtest from the BIT conventional; Wilson et al., 1987), a visual scanning test (i.e., the Picture scanning subtest from the BIT behavioral; Wilson et al., 1987), and a cancelation test (i.e., the Bells test; Gauthier et al., 1989). In addition, a non-spatial test (i.e., the Semantic verbal fluency test; Novelli et al., 1986) was also administered as a control test. The order of the daily-administered outcome measures was always the same (i.e., Picture scanning, Bells test, Line bisection, Semantic verbal fluency). The same examiner delivered all treatment sessions and she was aware of the aim of each treatment.

#### Picture scanning test

On this test, three large photographs were presented to the patient, one at a time (Wilson et al., 1987). The photographs depicted: a meal, a wash basin and toiletries, and a large hospital room containing various pieces of furniture and hospital aids. The midline of each photograph was aligned with the body midline of GR. He was asked to name the items in each photograph. Omissions of items were scored. There was no time limit for the patient to perform the test.

#### Bells test

On this test, different black drawings (i.e., shadows) including 35 targets (bells) and 280 distractors were printed on a white A4 sheet of paper (210 mm× 297 mm) (Gauthier et al., 1989). The drawings were positioned in an apparently random order, but they were equally distributed in seven columns (three on the left, three on the right, and one central), numbered from one to seven starting from the left. The midline of the A4 sheet of paper was aligned with the body midline of the patient. GR was asked to sign with a circle the targets (bells) in the A4 sheet of paper. Omissions of targets were scored. There was no time limit for the patient to perform the test.

#### Line bisection

The test consisted of three, 20-cm-long, horizontal, black line segments, one placed on the right side, one on the center, and one on the left side of a white A4 sheet of paper (210 mm × 297 mm) (Wilson et al., 1987). The midline of the A4 sheet was aligned with the body midline of the patient. GR was asked to find and mark the center of each line segment. The distance of the mark from each midline was measured. For each mark, a score from 0 (high displacement) to 3 (low displacement) was assigned according to the correction sheet. There was no time limit for the patient to perform the test.

#### Semantic verbal fluency test

On this test, GR was required to orally produce the highest possible number of words belonging to three semantic categories: car brands, fruits, and animals (Novelli et al., 1986). GR had 1 min to produce the names from each semantic category. Each correctly produced name received one point.

### Stimuli

GR sat in front of a PC screen at a distance of about 60 cm. Stimuli comprised computerized exercises requiring simple and complex reaction times (http://www.schuhfried.com/cogniplus-cps/rehacom/), visuo-spatial word search exercises (De Tanti and Inzaghi, 1992), and visuo-spatial exercises in which the patient was asked to compare vertical bars presented at different distances. The vertical bars were moving at different speeds (De Tanti and Inzaghi, 1992). GR responded orally in the visual-search exercises, whereas he pressed a button with his right hand in the simple and complex reaction time exercises.

### Experimental design

**Figure 2:**
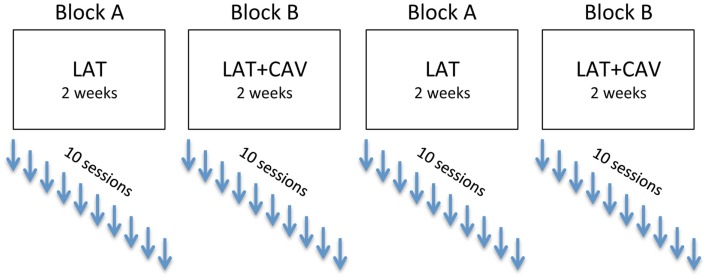
**Experimental ABAB design**. After each rehabilitation session, the patient was administered four neuropsychological tests (see Materials and Methods section for details).

An experimental ABAB blocks design was used: block A consisted of repeated sessions of LAT, whereas block B consisted of repeated sessions of LAT + CAV. The rehabilitation program (i.e., ABAB blocks) was completed approximately in 8 weeks. Each rehabilitation block consisted of 10 sessions of 1 h each, held once a day at the same hour (whenever possible), for 5 days a week (see Figure 2).

### Apparatus and rehabilitation procedure

The examiner sat behind GR, out of the patient’s sight, to avoid providing him with visual cues. During the rehabilitation sessions, the examiner prompted GR, whenever necessary, to carry out the computerized exercises. During the visual-search exercises and the visuo-spatial comparison of moving vertical bars, the examiner gave general verbal instructions to GR (e.g., “pay attention” or “check the stimuli in the whole visual field”), but avoided specific lateralized spatial suggestions (e.g., “pay attention to the left side of the screen” or “check the stimuli both on the left and on the right side of the visual field”). The exercises remained the same through the whole rehabilitation protocol and were presented in fixed-sequence order to GR.

#### Limb activation treatment

In the block A, the training involved the use of the “Limb Activation Treatment Device” (LAT-D), a modified version of the original Limb Activation Device (LAD), employed by Robertson et al. (2002). The LAT-D comprised a central unit and a bellows. The central unit encompassed a small plastic box, measuring 11 cm × 6 cm × 3 cm (weight = 150 g). The box contained the power supply, a microcontroller, a timer, a buzzer, and a LED. The control unit could activate a buzzer and display a light, at random or fixed intervals. The bellows (measuring 15.2 × 2.5 cm) could be pressed by GR to stop the buzzing tone emitted by the buzzer. The central unit was connected with the buzzer with a spiral plastic air tube, so as the distance between the box and the bellows could be easily adjusted. The bellows was fixed between the patient’s arm and the left armrest of the wheelchair. Then, the left arm of GR was placed on the bellows in order to compress it. By maintaining this setting, GR was asked to complete the computerized exercises. Each time GR heard the tone emitted by the buzzer, he was instructed to move his left arm to decompress the bellows to turn-off the tone. During the treatment, the buzzer was set to emit the tone at a fixed time interval of 120 s. If GR did not move his left arm within 30 s from the onset of the tone, the examiner verbally reminded him to move his left arm to decompress the bellows for turning-off the tone. This procedure remained the same through all the sessions of LAT. GR had sufficient proximal movement of his left arm to carry-on the rehabilitation protocol.

#### Contralateral arm vibration

The rehabilitation procedure of block B was the same as that of block A, except for the addition of a vibrating stimulus on the left, contralesional forearm of the patient. A portable vibration device (PVD) delivered the vibration. The device consisted of a small plastic unit, roughly 13 cm × 7 cm × 5 cm, with an elastomeric pressure-activated switch-pad, inside the PVD’s plastic body, and a clamping component that permitted us to fix the PVD on the patient’s left forearm. The PVD could be set up for the running time of vibration, for a fixed duration. The PVD remained attached on the left forearm of the patient during the entire rehabilitation session. The device was set to emit a constant vibration (frequency: ∼86 Hz) on the patient’s left forearm for a fixed time interval of 5 min. Among the fixed time intervals, a pause of 5 min was allowed to avoid the habituation of the patient’s forearm skin mechanical receptors. During the 5-min interval following PVD vibration, a sensation of “vibration aftereffect” was reported by GR. The procedure was the same for all the sessions of blocks B (i.e., LAT + CAV).

## Results

### The *C*-statistic analysis

We analyzed the data with the *C*-statistic test. The *C*-statistic is a statistical test that can be used to evaluate the trend in time-series measures, even when the number of observations is very low (e.g., at least eight observations for each experimental block; Young, 1941). By means of the *C*-statistic, the variability in successive data points is evaluated by examining the changes in slope from one block of an experiment to the next block of the same experiment (Tryon, 1982). In particular, the *C*-statistic estimates if, in a given dataset, there is a significant data trend. The *C*-statistic can be used to analyze separately each experimental block, but also it can be used to estimate if there are differences between successive blocks of the same experiment. To this aim, the data segments of the different blocks are joined in a unique vector (e.g., A + B, in an AB block design) and the statistical analysis on this joined vector is performed. In the present study, a significant *C*-statistic was considered as the evidence of a significant change between the different treatment blocks. To effectively use the *C*-statistic, a time-series of baseline scores is required. Given that multiple baseline scores were not available for GR, we assumed that the only baseline score available of GR could be a satisfying estimate of the patient’s condition. We thus created a vector by replicating 10 times (as for all the other experimental blocks) the value of the patient’s score at the baseline. Given this strong, but necessary assumption to use the *C*-statistic, we discussed the present results focused on the comparison between the treatment blocks (i.e., A and B), rather than on the comparisons between each treatment block and the baseline.

In the following analyses the experimental blocks have been labeled as follow: BAS is the baseline; LAT/1 is the first block of rehabilitation with LAT; LAT + CAV/1 is the first block of rehabilitation with LAT + CAV; LAT/2 is the second rehabilitation block with LAT; and LAT + CAV/2 is the second rehabilitation block with LAT + CAV. According to these labels, the treatment sequence was: BAS — LAT/1 — LAT + CAV/1 — LAT/2 — LAT + CAV/2 (see Figure 2). Separate *C*-statistics were calculated for all the tests administered (i.e., Picture scanning, Bells test, Line bisection, Semantic verbal fluency). Within each test, a *C*-statistic was calculated for each block, to investigate whether there was a significant trend within each block (i.e., LAT/1, LAT + CAV/1, LAT/2, or LAT + CAV/2). *C*-statistics were also calculated for each pair of consequent blocks, joined in a unique vector, to investigate whether there was a significant difference between two consequent blocks (i.e., BAS vs. LAT/1, LAT/1 vs. LAT + CAV/1, LAT + CAV/1 vs. LAT/2, LAT/2 vs. LAT + CAV/2).

### Results of the *C*-statistic analyses

**Figure 3:**
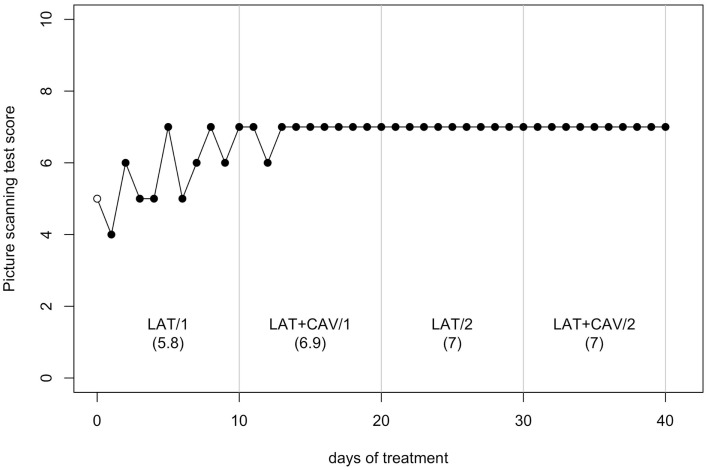
**Trend of GR’s performance on the Picture scanning test**. The first data point (empty dot) indicates the baseline score (5). The values under each block label indicate GR’s mean score of the 10 sessions composing each block.

The *C*-statistic analysis of the data from the Picture scanning test (Wilson et al., 1987) showed a significant trend between the LAT/1 and the LAT + CAV/1 blocks [analysis on LAT/1 vs. LAT + CAV/1 vector, *C* = 0.42, *z* = 1.81, *p* < 0.05; LAT/1 mean = 5.8, LAT + CAV/1 mean = 6.35]. No other significant trends were found within or between blocks (all *p*s > 0.05) – (see Figure 3). Although a significant difference was found between the LAT/1 and the LAT + CAV/1 blocks, it is impossible to attribute the improvement observed to an effect of the combined treatments (i.e., LAT + CAV) because of the absence of subsequent variability of GR’s performance.

**Figure 4:**
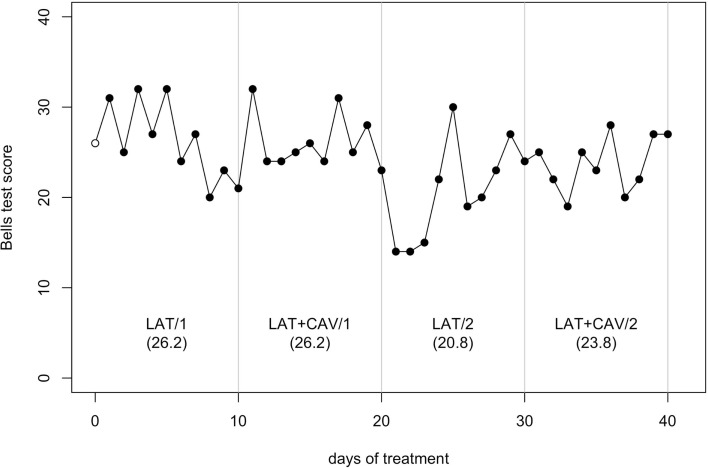
**Trend of GR’s performance on the Bells test**. The first data point (empty dot) indicates the baseline score (26). The values under each block label indicate GR’s mean score of the 10 sessions composing each block.

The *C*-statistic analysis of the data from the Bells test (Gauthier et al., 1989) showed a significant trend between the LAT + CAV/1 and the LAT/2 blocks [analysis on the LAT + CAV/1 vs. LAT/2 vector, *C* = 0.46, *z* = 1.97, *p* < 0.05; LAT + CAV/1 mean = 26.2, LAT/2 mean = 20.8] and between the LAT/2 and the LAT + CAV/2 blocks [analysis on the LAT/2 vs. LAT + CAV/2 vector, *C* = 0.44, *z* = 1.88, *p* < 0.05; LAT/2 mean = 20.8, LAT + CAV/2 mean = 22.3] – (see Figure 4). Thus, GR’s performance on the Bells test was better after the application of the combined treatments (i.e., LAT + CAV), rather than after the LAT alone. Although the mean score in the LAT1 condition was 26.1, a trend within this condition was not found. Given the absence of a meaningful baseline, it is impossible to infer the presence of an improvement in this condition with respect to the baseline.

**Figure 5:**
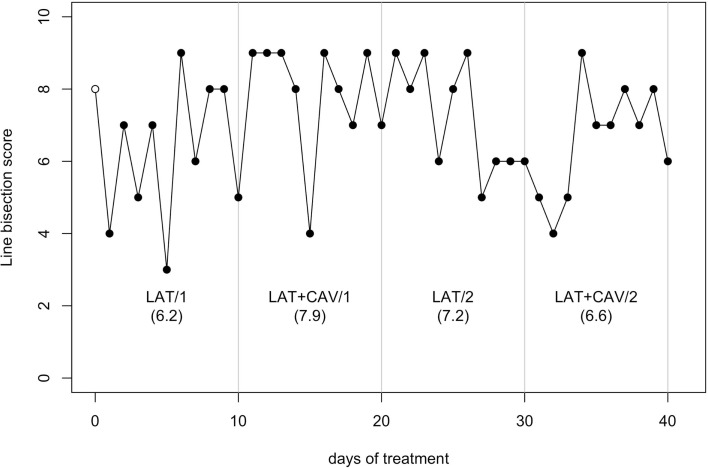
**Trend of GR’s performance on the Line bisection test**. The first data point (empty dot) indicates the baseline score (8). The values under each block label indicate GR’s mean score of the 10 sessions composing each block.

The *C*-statistic analysis of the data from the Line bisection (Wilson et al., 1987) showed no significant trend neither in between blocks comparisons, nor in within-block comparisons (all *p*s > 0.05; see Figure 5).

**Figure 6:**
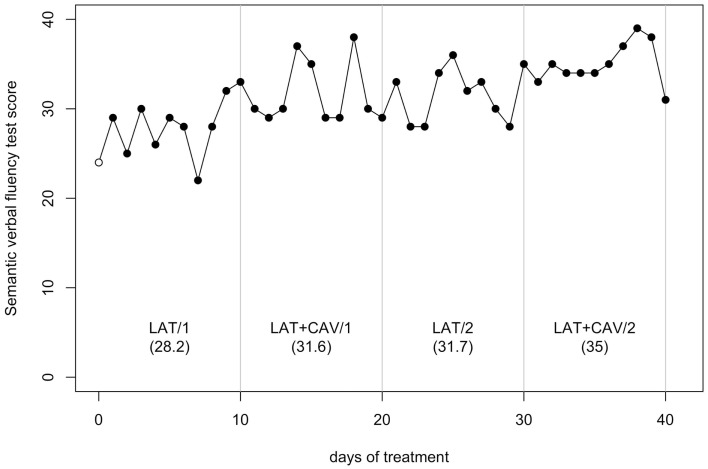
**Trend of GR’s performance on the Semantic verbal fluency test**. The first data point (empty dot) indicates the baseline score (24). The values under each block label indicate GR’s mean score of the 10 sessions composing each block.

The *C*-statistic analysis of the data from the Semantic verbal fluency test (Novelli et al., 1986) showed a significant difference between the BAS and the LAT/1 block [analysis on the BAS + LAT/1 vector, *C* = 0.51, *z* = 2.16, *p* < 0.05; BAS = 24, LAT/1 mean = 28.2] and a significant difference between the LAT/2 and the LAT + CAV/2 blocks [analysis on the LAT/2 — LAT + CAV/2 vector, *C* = 0.44, *z* = 1.86, *p* < 0.05; LAT/2 mean = 31.7, LAT + CAV/2 = 35]; see Figure 6.

## Discussion

We studied GR, a patient who initially suffered a right-hemisphere stroke and then a left-hemisphere stroke. Following the right-hemisphere stroke, GR presented with severe LN. After his left-hemisphere stroke, which was limited to the lenticular nucleus, we did not observe any further behavioral changes of GR, except of a temporary presence of speech apraxia. Indeed, he had no linguistic deficits in everyday life and on tests that require oral verbal comprehension and production (e.g., the MMSE and the Verbal reasoning test). Approximately 2-months after GR’s left-hemisphere stroke, the present ABAB rehabilitation study started.

We tested the possible additive effects of two rehabilitation techniques (i.e., the LAT and the CAV). By using these techniques, we aimed to prompt the activation of the lesioned right hemisphere. The LAT was used as an *active* (i.e., mainly top-down) limb activation technique, whereas the CAV was used as a *passive* (i.e., bottom-up) tactile activation technique. GR showed high IIV in his performance on visuo-spatial tests. Some aspects of what is interpreted as change may be, therefore, attributable to short-term fluctuation and sampling variation, rather than true change (Salthouse, 2007). The success of an intervention, indeed, may not be evident because IIV and other types of variables (e.g., medical therapies, physiotherapy, unspecific environmental stimulation, etc.) might not have been appropriately considered. Thus, given the high IIV of GR, an ABAB repeated-measures design was used. The clearest of our results was that the combined application of LAT and CAV induced an improvement of GR’s performance on the Bells test (Gauthier et al., 1989). This finding suggests that the amelioration of GR’s performance could be the consequence of a specific sensori-motor activation effect of the right hemisphere after the combined activation (i.e., active with LAT and passive with CAV) of the contralesional left arm. It is, then, possible that the activation of the left contralesional arm in the left space has enhanced the neural networks that subserve space representation in the lesioned right hemisphere.

GR’s performance on the Bells test got worse specifically from LAT + CAV/1 to LAT/2. That is, when CAV was not applied anymore, GR’s performance got worse, whereas when CAV was re-applied GR’s performance was improved again. Finally, there were no intra-block differences in GR’s performance on the Bells test. Taken together, these findings are in favor of a specific sensori-motor effect of LAT + CAV on the damaged circuits of the right hemisphere. A limitation of the present study, however, should be underlined. Given the lack of repeated measures on the baseline of GR, all results should be taken cautiously, and further studies with measures on baseline are necessary to corroborate the present results.

The positive effects of LAT + CAV were limited on one test measuring LN signs (i.e., the Bells test). In contrast, there were no positive effects of LAT + CAV on the other two LN tests (i.e., Picture scanning test and Line bisection test). Note, however, that cancelation and bisection tasks are doubly dissociated in neurological patients (Halligan and Marshall, 1992; Keller et al., 2005). As a consequence, different rehabilitation techniques might be required to yield positive effects also on line bisection tasks. Finally, an important procedural difference between the Bells test and the Picture scanning test should be noted. On the Bells test, patients are required to perform actions with their ipsilesional limb toward the ipsi- and the contralesional side of space. In contrast, on the Picture scanning test no actions are required, given that patients are asked to verbally describe a picture placed in front of them. This limb-motor vs. verbal-motor output difference should be further investigated in future studies, given that these aspects of LN are doubly dissociated (e.g., see Heilman et al., 2003).

If our findings were a consequence of generalized and unspecific brain activation, we would have found exactly the same trend of amelioration, as that observed on the Bells test, also on the Semantic verbal fluency test. In contrast, GR’s performance on the control task ameliorated only from LAT/2 to LAT + CAV/2. Thus, generalized and unspecific brain activation might explain GR’s performance improvement from LAT/2 to LAT + CAV/2, but cannot explain GR’s performance deterioration from LAT + CAV/1 to LAT/2. Note, however, that if the improvement in GR’s performance between LAT/2 and LAT + CAV/2 was only due to generalized and unspecific brain activation, GR’s performance amelioration would have been observed on all tests. This was not the case.

Karnath (1995) used the CAV as an experimental control task, with four LN patients who were asked to perform a cancelation and a copying task. He found no improvement on patients’ performance on the two tasks following the CAV. There are, however, some methodological differences between our study and that of Karnath. First, in the Karnath’s study the sequence of blocks was not counterbalanced (CAV was always applied in the last block), whereas we used an ABAB design. Second, Karnath applied CAV on the left hand of each patient, whereas we applied CAV on GR’s left forearm. Third, Karnath applied CAV for a very brief duration (i.e., during the execution of cancelation and copying tasks), whereas we applied the CAV for 30′ on each LAT + CAV rehabilitation session, for 10 consecutive sessions. Finally, Karnath used the CAV alone, whereas we used a combination of LAT and CAV to reach a more enhanced activation of the sensori-motor circuits of the right lesioned hemisphere.

There is considerable evidence in the literature on the effectiveness of LAT for some LN patients (Robertson and North, 1992, 1993, 1994; Robertson et al., 1992, 1998; Samuel et al., 2000; Bailey et al., 2002). Nonetheless, the previous results are far from being clear because of the different methodologies used and the different neuropsychological measures adopted. In the present study, a reliable assessment of GR’s performance was very difficult because of his high IIV. Our preliminary positive results might provide some new evidence on the possibility to obtain additive effects of cognitive rehabilitation procedures, if these procedures are based on a common theoretical framework and, consequently, share a common network of neural activation subserving the target function. The present findings suggest the need of more extensive LN rehabilitation studies that combine multiple treatments, by means of the application of theory-driven cognitive interventions. Although the simultaneous application of the LAT and the CAV, together with the use of a repeated-measures design (e.g., ABAB) is promising, future single case and group studies are needed to examine in depth the effects of the LAT combined with the CAV in order to reduce LN signs.

## Conflict of Interest Statement

The authors declare that the research was conducted in the absence of any commercial or financial relationships that could be construed as a potential conflict of interest.

## References

[B1] Adair J. C. Barrett A. M. (2008). Spatial neglect: clinical and neuroscience review. A wealth of information on the poverty of spatial attention. Ann. N. Y. Acad. Sci. 1142, 21–43 10.1196/annals.1444.008 18990119 PMC2962986

[B2] Antonucci G. Guariglia C. Judica A. Magnotti L. Paolucci S. Pizzamiglio L. (1995). Effectiveness of neglect rehabilitation in a randomized group study. J. Clin. Exp. Neuropsychol. 17, 383–389 10.1080/01688639508405131 7650101

[B3] Bailey M. J. Riddoch M. J. Crome P. (2002). Treatment of visual neglect in elderly patients with stroke: a single-subject series using either a scanning and cueing strategy or a left-limb activation strategy. Phys. Ther. 82, 782–797 12147008

[B4] Barrett A. M. Buxbaum L. J. Coslett H. B. Edwards E. Heilman K. M. Hillis A. E. (2006). Cognitive rehabilitation interventions for neglect and related disorders: moving from bench to bedside in stroke patients. J. Cogn. Neurosci. 18, 1223–1236 10.1162/jocn.2006.18.7.1223 16839294

[B5] Butter C. M. Kirsch N. (1992). Combined and separate effects of eye patching and visual stimulation on unilateral neglect following stroke. Arch. Phys. Med. Rehabil. 73, 1133–1139 1463376

[B6] Buxbaum L. J. Ferraro M. K. Veramonti T. Farnè A. Whyte J. Làdavas E. (2004). Hemispatial neglect: subtypes, neuroanatomy, and disability. Neurology 62, 749–756 10.1212/01.WNL.0000113730.73031.F4 15007125

[B7] Carlesimo G. A. Caltagirone C. Gainotti G. (1996). The Mental Deterioration Battery: normative data, diagnostic reliability and qualitative analyses of cognitive impairment. The Group for the Standardization of the Mental Deterioration Battery. Eur. Neurol. 36, 378–384 10.1159/000117297 8954307

[B8] Cherney L. R. Halper A. S. Kwasnica C. M. Harvey R. L. Zhang M. (2001). Recovery of functional status after right hemisphere stroke: relationship with unilateral neglect. Arch. Phys. Med. Rehabil. 82, 322–328 10.1053/apmr.2001.21511 11245753

[B9] Cubelli R. Paganelli N. Achilli D. Pedrizzi S. (1999). Is one hand always better than two? A replication study. Neurocase 5, 143–151 10.1080/13554799908415478

[B10] De Tanti A. Inzaghi M. G. (1992). Pong: Programmi per la Riabilitazione Cognitiva della Negligenza Spaziale Unilaterale. Como: Fumagalli Ricerca e Cultura

[B11] Denes G. Semenza C. Stoppa E. Lis A. (1982). Unilateral spatial neglect and recovery from hemiplegia: a follow-up study. Brain 105, 543–552 10.1093/brain/105.3.543 7104665

[B12] Farnè A. Buxbaum L. Ferraro M. Frassinetti F. Whyte J. Veramonti T. (2004). Patterns of spontaneous recovery of neglect and associated disorders in acute right brain-damaged patients. J. Neurol. Neurosurg. Psychiatr. 75, 1401–1410 10.1136/jnnp.2002.003095 15377685 PMC1738754

[B13] Ferber S. Bahlo S. Ackermann H. Karnath H.-O. (1998). Vibration der nackenmuskulatur als therapie bei neglectsymptomatik? Eine fallstudie. Neurol. Rehabil. 4, 21–24

[B14] Frassinetti F. Rossi M. Làdavas E. (2001). Passive limb movements improve visual neglect. Neuropsychologia 39, 725–733 10.1016/S0028-3932(00)00156-1 11311302

[B15] Gauthier L. Dehaut F. Joanette Y. (1989). The Bells test: a quantitative and qualitative test for visual neglect. Int. J. Clin. Neuropsychol. 11, 49–54

[B16] Halligan P. W. Manning L. Marshall J. C. (1991). Hemispheric activation vs. spatio-motor cueing in visual neglect: a case study. Neuropsychologia 29, 165–176 10.1016/0028-3932(91)90018-4 2027432

[B17] Halligan P. W. Marshall J. C. (1992). Left visuo-spatial neglect: a meaningless entity? Cortex 28, 525–535 10.1016/S0010-9452(13)80225-0 1478083

[B18] Harding P. Riddoch M. J. (2009). Functional electrical stimulation (FES) of the upper limb alleviates unilateral neglect: a case series analysis. Neuropsychol. Rehabil. 19, 41–63 10.1080/09602010701852610 18609022

[B19] Heilman K. M. Watson R. T. Valenstein E. (2003). “Neglect and related disorders,” in Clinical Neuropsychology, 4th Edn, eds Heilman K. M. Valenstein E. (New York: Oxford University Press), 243–293

[B20] Humphreys G. W. Watelet A. Riddoch M. J. (2006). Long-term effects of prism adaptation in chronic visual neglect: a single case study. Cogn. Neuropsychol. 23, 463–478 10.1080/02643290500202755 21049340

[B21] Jehkonen M. Ahonen J. P. Dastidar P. Koivisto A. M. Laippala P. Vilkki J. (2000). Visual neglect as a predictor of functional outcome one year after stroke. Acta Neurol. Scand. 101, 195–201 10.1034/j.1600-0404.2000.101003195.x 10705943

[B22] Jehkonen M. Ahonen J. P. Dastidar P. Koivisto A. M. Laippala P. Vilkki J. (2001). Predictors of discharge to home during the first year after right hemisphere stroke. Acta Neurol. Scand. 104, 136–141 10.1034/j.1600-0404.2001.00025.x 11551232

[B23] Jehkonen M. Laihosalo M. Koivisto A. M. Dastidar P. Ahonen J. P. (2007). Fluctuation in spontaneous recovery of left visual neglect: a 1-year follow-up. Eur. Neurol. 58, 210–214 10.1159/000107941 17823534

[B24] Johannsen L. Ackermann H. Karnath H.-O. (2003). Lasting amelioration of spatial neglect by treatment with neck muscle vibration even without concurrent training. J. Rehabil. Med. 35, 249–253 10.1080/16501970310009972 14664313

[B25] Karnath H. O. (1995). Transcutaneous electrical stimulation and vibration of neck muscles in neglect. Exp. Brain Res. 105, 321–324 10.1007/BF00240969 7498386

[B26] Karnath H.-O. Christ K. Hartje W. (1993). Decrease of contralateral neglect by neck muscle vibration and spatial orientation of trunk midline. Brain 116, 383–396 10.1093/brain/116.2.383 8461972

[B27] Katz N. Hartman-Maeir A. Ring H. Soroker N. (1999). Functional disability and rehabilitation outcome in right hemisphere damaged patients with and without unilateral spatial neglect. Arch. Phys. Med. Rehabil. 80, 379–384 10.1016/S0003-9993(99)90273-3 10206598

[B28] Keller I. Lefin-Rank G. Lösch J. Kerkhoff G. (2009). Combination of pursuit eye movement training with prism adaptation and arm movements in neglect therapy: a pilot study. Neurorehabil. Neural Repair 23, 58–66 10.1177/1545968308317438 18801912

[B29] Keller I. Schindler I. Kerkhoff G. Von Rosen F. Golz D. (2005). Visuospatial neglect in near and far space: dissociation between line bisection and letter cancellation. Neuropsychologia 43, 724–731 10.1016/j.neuropsychologia.2004.08.003 15721185

[B30] Kerkhoff G. Schenk T. (2012). Rehabilitation of neglect: an update. Neuropsychologia 50, 1072–1079 10.1016/j.neuropsychologia.2012.01.024 22306520

[B31] Lafosse C. Kerckhofs E. Troch M. Vandenbussche E. (2003). Upper limb exteroceptive somatosensory and proprioceptive sensory afferent modulation of hemispatial neglect. J. Clin. Exp. Neuropsychol. 25, 308–323 10.1076/jcen.25.3.308.13807 12916645

[B32] Luauté J. Halligan P. Rode G. Rossetti Y. Boisson D. (2006). Visuo-spatial neglect: a systematic review of current interventions and their effectiveness. Neurosci. Biobehav. Rev. 30, 961–982 10.1016/j.neubiorev.2006.03.001 16647754

[B33] Maddicks R. Marzillier S. L. Parker G. (2003). Rehabilitation of unilateral neglect in the acute recovery stage: the efficacy of limb activation therapy. Neuropsychol. Rehabil. 13, 391–408 10.1080/09602010244000264 21854320

[B34] Magni E. Binetti G. Padovani A. Cappa S. F. Bianchetti A. Trabucchi M. (1996). The mini-mental state examination in Alzheimer’s disease and multi-infarct dementia. Int. Psychogeriatr. 8, 127–134 10.1017/S1041610296002529 8805093

[B35] Nijboer T. C. W. Kollen B. J. Kwakkel G. (in press). Time course of visuospatial neglect early after stroke: a longitudinal cohort study. Cortex. 10.1016/j.cortex.2012.11.006 23332473

[B36] Novelli G. Papagno C. Capitani E. Laiacona M. Vallar G. Cappa S. F. (1986). Tre test clinici di ricerca e produzione lessicale. Taratura su sogetti normali. Arch. Psicol. Neurol. Psichiatr. 47, 477–506

[B37] Nudo R. J. McNeal D. (2013). “Plasticity of cerebral functions,” in Handbook of Clinical Neurology (3rd Series), eds Barnes M. P. Good D. C. (Amsterdam: Elsevier), 13–21 10.1016/B978-0-444-52901-5.00002-2 23312627

[B38] Pizzamiglio L. Fasotti L. Jehkonen M. Antonucci G. Magnotti L. Boelen D. (2004). The use of optokinetic stimulation in rehabilitation of the hemineglect disorder. Cortex 40, 441–450 10.1016/S0010-9452(08)70138-2 15259325

[B39] Polanowska K. Seniów J. Paprot E. Lesniak M. Czlonkowska A. (2009). Left-hand somatosensory stimulation combined with visual scanning training in rehabilitation for post-stroke hemineglect: a randomised, double-blind study. Neuropsychol. Rehabil. 19, 364–382 10.1080/09602010802268856 18663642

[B40] Rengachary J. He B. J. Shulman G. L. Corbetta M. (2011). A behavioral analysis of spatial neglect and its recovery after stroke. Front. Hum. Neurosci. 5:29 10.3389/fnhum.2011.00029 21519374 PMC3075878

[B41] Riddoch M. J. Humphreys G. W. (1983). The effect of cueing on unilateral neglect. Neuropsychologia 21, 589–599 10.1016/0028-3932(83)90056-8 6664478

[B42] Riestra A. R. Barrett A. M. (2013). “Rehabilitation of spatial neglect,” in Handbook of Clinical Neurology (3rd Series), eds Barnes M. P. Good D. C. (Amsterdam: Elsevier), 347–355 10.1016/B978-0-444-52901-5.00029-0 PMC3988490 23312654

[B43] Rizzolatti G. Berti A. (1990). Neglect as a neural representation deficit. Rev. Neurol. (Paris) 146, 626–634 2124720

[B44] Rizzolatti G. Camarda R. (1987). “Neural circuits for spatial attention and unilateral neglect,” in Neurophysiological and Neuropsychological Aspects of Spatial Neglect, ed. Jeannerod M. (Amsterdam: North Holland), 289–313

[B45] Robertson I. H. Hogg K. McMillan T. M. (1998). Rehabilitation of visual neglect: improving function by contralesional limb activation. Neuropsychol. Rehabil. 8, 19–29 10.1080/713755556

[B46] Robertson I. H. McMillan T. M. MacLeod E. Edgeworth J. Brock D. (2002). Rehabilitation by limb activation training reduces left-sided motor impairment in unilateral neglect patients: a single-blind randomised control trial. Neuropsychol. Rehabil. 12, 439–454 10.1080/09602010244000228

[B47] Robertson I. H. North N. T. (1992). Spatiomotor cueing in unilateral neglect: the role of hemispace, hand and motor activation. Neuropsychologia 30, 553–563 10.1016/0028-3932(92)90058-T 1641119

[B48] Robertson I. H. North N. T. (1993). Active and passive activation of left limbs: influence on visual and sensory neglect. Neuropsychologia 31, 293–300 10.1016/0028-3932(93)90093-F 8492882

[B49] Robertson I. H. North N. T. (1994). One hand is better than two: motor extinction of left hand advantage in unilateral neglect. Neuropsychologia 32, 1–11 10.1016/0028-3932(94)90064-7 8818150

[B50] Robertson I. H. North N. T. Geggie C. (1992). Spatiomotor cueing in unilateral left neglect: three case studies of its therapeutic effect. J. Neurol. Neurosurg. Psychiatr. 55, 799–805 10.1136/jnnp.55.9.799 1402971 PMC1015105

[B51] Saevarsson S. Halsband U. Kristjánsson Á (2011). Designing rehabilitation programs for neglect: could 2 be more than 1+1? Appl. Neuropsychol. 18, 95–106 10.1080/09084282.2010.547774 21660761 PMC4544767

[B52] Saevarsson S. Kristjánsson Á. Halsband U. (2010). Strength in numbers: combining neck vibration and prism adaptation produces additive therapeutic effects in unilateral neglect. Neuropsychol. Rehabil. 20, 704–724 10.1080/09602011003737087 20503132 PMC3129649

[B53] Salthouse T. A. (2007). Implications of within-person variability in cognitive and neuropsychological functioning for the interpretation of change. Neuropsychology 21, 401–411 10.1037/0894-4105.21.4.401 17605573 PMC3838958

[B54] Samuel C. Louis-Dreyfus A. Kaschel R. Makiela E. Troubat M. Anselmi N. (2000). Rehabilitation of very severe unilateral neglect by visuo-spatio-motor cueing: two single case studies. Neuropsychol. Rehabil. 10, 385–399 10.1080/096020100411970

[B55] Schindler I. Kerkhoff G. Karnath H.-O. Keller I. Goldenberg G. (2002). Neck muscle vibration induces lasting recovery in spatial neglect. J. Neurol. Neurosurg. Psychiatr. 73, 412–419 10.1136/jnnp.73.4.412 12235310 PMC1738082

[B56] Schröder A. Wist E. R. Hömberg V. (2008). TENS and optokinetic stimulation in neglect therapy after cerebrovascular accident: a randomized controlled study. Eur. J. Neurol. 15, 922–927 10.1111/j.1468-1331.2008.02229.x 18637956

[B57] Sharma N. Classen J. Cohen L. G. (2013). “Neural plasticity and its contribution to functional recovery,” in Handbook of Clinical Neurology (3rd Series), eds Barnes M. P. Good D. C. (Amsterdam: Elsevier), 3–12 10.1016/B978-0-444-52901-5.00001-0 PMC4880010 23312626

[B58] Spinnler H. Tognoni G. (1987). Standardizzazione e taratura di test neuropsicologici. Ital. J. Neurol. Sci. 8(Suppl.), 6

[B59] Stone S. P. Patel P. Greenwood R. J. Halligan P. W. (1992). Measuring visual neglect in acute stroke and predicting its recovery: the visual neglect recovery index. J. Neurol. Neurosurg. Psychiatry 55, 431–436 10.1136/jnnp.55.6.431 1619406 PMC1014895

[B60] Stuss D. T. (2011). The future of cognitive neurorehabilitation. Neuropsychol. Rehabil. 21, 755–768 10.1080/09602011.2011.605590 21950776

[B61] Tryon W. W. (1982). A simplified time-series analysis for evaluating treatment interventions. J. Appl. Behav. Anal. 15, 423–429 10.1901/jaba.1982.15-423 7142062 PMC1308286

[B62] Vallar G. (1998). Spatial hemineglect in humans. Trends Cogn. Sci. (Regul. Ed.) 2, 87–97 10.1016/S1364-6613(98)01145-0 21227084

[B63] Vallar G. Rusconi M. L. Barozzi S. Bernardini B. Ovadia D. Papagno C. (1995). Improvement of left visuo-spatial hemineglect by left-sided transcutaneous electrical stimulation. Neuropsychologia 33, 73–82 10.1016/0028-3932(94)00088-7 7731542

[B64] Wilson B. Cockburn J. Halligan P. W. (1987). The Behavioural Inattention Test. Bury St. Edmunds: Thames Valley Test Company

[B65] Young L. C. (1941). On randomness in ordered sequences. Ann. Math. Stat. 12, 293–300 10.1214/aoms/1177731711

